# Stereotactic body radiotherapy (SBRT) for locally advanced intrahepatic and extrahepatic cholangiocarcinoma

**DOI:** 10.1186/s12885-017-3788-1

**Published:** 2017-11-21

**Authors:** Eleni Gkika, Lukas Hallauer, Simon Kirste, Sonja Adebahr, Nico Bartl, Hannes Philipp Neeff, Ralph Fritsch, Volker Brass, Ursula Nestle, Anca Ligia Grosu, Thomas Baptist Brunner

**Affiliations:** 1 Department of Radiation Oncology, University Medical Centre Freiburg, Freiburg im Breisgau, Germany; 2 Department of General and Visceral Surgery, University Medical Centre Freiburg, Freiburg im Breisgau, Germany; 3 Department of Internal Medicine, Haematology, Oncology and Stem-Cell Transplantation, University Medical Centre Freiburg, Freiburg im Breisgau, Germany; 4 Department of Gastroenterology, Hepatology, Endocrinology and Infectious Diseases, University Medical Centre Freiburg, Freiburg im Breisgau, Germany; 5 German Cancer Consortium (DKTK), partner site Freiburg, Heidelberg, Germany; 6 German cancer Research Centre (DKFZ), Heidelberg, Germany; 7 Faculty of Medicine, University of Freiburg, Freiburg im Breisgau, Germany

**Keywords:** SBRT, Stereotactic body radiotherapy, Intrahepatic and extrahepatic cholangiocarcinoma

## Abstract

**Background:**

To evaluate the role of ablative radiotherapy doses in the treatment of hilar or intrahepatic cholangiocarcinoma (CCC) using stereotactic body radiotherapy (SBRT).

**Methods:**

Consecutive patients treated from 2007 to 2016 with CCC were evaluated. Local control and toxicities were assessed every 3 months according to the Response Evaluation Criteria In Solid Tumors (RECIST) and the Common Terminology Criteria for Adverse Events v4.0, respectively. Overall survival (OS), local control (LC) and progression free survival were calculated from SBRT.

**Results:**

Thirty seven patients with 43 lesions were retrospectively evaluated. The median dose delivered was 45 Gy (range 25-66 Gy) in 3-12 fractions, corresponding to a median equivalent dose in 2 Gy fractions (EQD2_10_) of 56 (range 25-85) Gy. The median follow up was 24 months. The OS at 1 year was 56% with a median OS of 14 (95% CI: 7.8-20.2) months from start of SBRT and 22 (95% CI: 17.5-26.5) months from diagnosis. Eight lesions progressed locally. The local control rate (LC) at 1 year was 78%. The median progression free survival was 9 months (95% CI 2.8-15.2) 21 patients progressed in the liver but out of field and 15 progressed distantly. SBRT was well tolerated. Three patients (9%) developed a Grade III bleeding. Seven patients developed a cholangitis, one due to progression and the other because of a stent dysfunction 2-21(median 8) months from SBRT.

**Conclusion:**

In patients with locally advanced cholangiocarcinoma, SBRT is a local treatment option with an acceptable toxicity profile which warrants further investigation in prospective trials.

## Background

Cholangiocarcinoma (CCC) is the second most common primary liver cancer after hepatocellular carcinoma and is divided anatomically into intrahepatic (IHCC) and extrahepatic cholangiocarcinoma (EHCC). EHCCs are subdivided into hilar/perihilar (pCC, also called Klatskin tumors), or distal (dCC). pCC is the most common type of cholangiocarcinoma, followed by dCC and intrahepatic forms [1]. The only potentially curative treatment option is surgical resection but 70% of the patients are deemed irresectable [2] and about half of the patients undergoing resection relapse within 1 year after resection [3]. The current standard of care for both locally advanced and metastatic patients with good performance status is combination chemotherapy with platinum and gemcitabine-containing protocols, which achieve a median overall survival of 11.7 and a median progression free survival of 8 months [4]. Currently primary treatment options for patients with unresectable or metastatic disease according to the National Comprehensive Cancer Network (NCCN) Guidelines version 1.2017 include: 1) clinical trial; 2) fluoropyrimidine-based or gemcitabine-based chemotherapy; or 3) best supportive care. In addition, fluoropyrimidine chemoradiation is included as an option for patients with unresectable disease. Locoregional therapies such as radiofrequency ablation (RFA) [5], trans-arterial chemoembolization (TACE) [6], drug-eluting bead trans-arterial chemoembolization (DEB-TACE) or TACE drug-eluting microspheres [7] and transarterial radioembolization (TARE) with yttrium-90 microspheres [8] have been shown to be safe and effective in a small retrospective series of patients with unresectable intrahepatic cholangiocarcinomas (NCCN version 1.2017, hepatobiliary cancers). Hepatic arterial infusion (HAI) chemotherapy also was used for the treatment of patients with advanced and unresectable intrahepatic cholangiocarcinoma [9]. Furthermore, liver transplantation was used in selected patients with locally advanced hilar cholangiocarcinomas [10] and the combination of photodynamic therapy (PDT) with biliary stenting was reported to be associated with prolonged OS in patients with unresectable cholangiocarcinoma in small randomized clinical trials [11].

The role of radiotherapy remains controversial due to lack of phase III randomised trials. However, several early phase studies have suggested that radiotherapy can prolong survival [12, 13], and there is evidence of a dose response relationship [12, 14]. This has prompted the investigation of SBRT as a method for dose-escalation. SBRT is an emerging treatment technique for cholangiocarcinoma where ablative doses can be applied with a steep dose gradient and thus sparing normal tissue. SBRT can lead to high local control rates with moderate toxicity in other primary or metastatic cancers of the liver [15, 16].

In this study, we evaluated the role of SBRT in the treatment of CCC in respect to toxicity as well as local control.

## Methods

### Patients

After institutional review board approval we retrospectively analysed 37 consecutive patients treated at our centre with SBRT, either for positive margins after resection or for inoperable or recurrent, locally advanced CCC. All patients included in the analysis underwent multidisciplinary evaluation by medical, surgical and radiation oncologists.

Patients underwent clinical examinations and routinely laboratory tests before treatment and at least weekly during treatment, by the radiation oncologists of the department. During follow up, physical examination, blood tests and computed tomography (CT) or magnetic resonance imaging (MRI) were acquired every 3 months. Toxicity was scored using the NCI Common Terminology Criteria for Adverse Events v4.0. (National Cancer Institute: Common Terminology Criteria for Adverse Events Version 4.03, CTCAE 2010). All toxicities that were observed within 90 days after treatment were considered to be acute; all other toxicities reported after >90 days were considered to be late.

Primary end points were toxicity and local control (LC) in the planning target volume (PTV, or ‘in-field’) at 1 year; the latter was defined as the absence of progressive disease within the PTV as per Response Evaluation Criteria in Solid Tumors (RECIST) v1.1 [17]. Secondary end points were overall survival and patterns of failure. Lesions that progressed outside the PTV in the liver or lymph nodes were scored as regional progression and those developed in other organs as distant progression.

### SBRT techniques

Patients were immobilized in supine position with a customized vacuum cushion using abdominal compression to minimize respiratory motion and underwent a 4 dimensional-CT (4D CT) or 4D fluorodeoxyglucose positron emission tomography (FDG PET/CT), as described elsewhere [18]. The gross tumor volume (GTV) was defined based on available imaging including the finding of the Endoscopic Retrograde Cholangiopancreatography (ERCP), Magnetic Resonance Cholangiopancreatography (MRCP), MRI, CT and/or PET CT. The internal target volume (ITV) was created accounting for the extent and the position of the tumour at all motion phases in 3 dimensions of the 4D-CT or 4D-PET/CT. The PTV was a uniform 4 mm expansion of the ITV in all dimensions. Organs at risk (OAR) included heart, liver, lung, ribs, skin, spinal cord, stomach, small intestine, colon, duodenum, kidneys, bile duct and large vessels were defined as applicable. Dose constraints were defined according to Timmerman et al. [19].Patients were treated with 3 to 12 fractions delivered every other day, depending on the proximity to OARs, mostly the stomach and the intestine. Three fraction regimens (typically 3 × 12.5Gy) were preferred in patients with lesions at distance from critical structures, 12 fraction regimens (typically 12 × 4-5.5 Gy) were preferred in patients with direct contact to OARs, and 5 fraction regimens (typically 5 × 7-10 Gy) in all other cases, so that the dose constraints could be respected. From 2007 to 2013 treatment was prescribed either to the 60% or 80% encompassing isodose and thereafter according to ICRU report 83. For lesions where dose constraints for the OARs could not be achieved, we used a simultaneous integrated protection (SIP) dose prescription, instead of reducing the dose to the entire PTV. The SIP approach is an intensity modulated radiotherapy (IMRT) technique described in detail elsewhere [20]. For the analysis, the prescribed doses, as well as the maximum and mean dose delivered were converted to equieffective doses for 2 Gy fractions (EQD2), assuming that tumour, late reacting bowel tissue and liver α/β ratios were 10 Gy, 3 Gy and 2 Gy, respectively [21].

For all patients a daily on-line correction using cone beam computed tomography (CBCT) scans was applied and oral contrast was given to visualise stomach and/or duodenum in cases of close proximity.

### Statistical analysis

Descriptive statistics were used to analyse patient, tumor and treatment characteristics. Survival and control times were calculated from the start of SBRT. Time to progression and survival were assessed using the Kaplan-Meier method and the Cox proportional hazards model. Analyses were performed using SPSS (SPSS Inc., Chicago, IL) Statistical significance was set to *p* ≤ .05 and two sided.

## Results

### Patient, tumor and treatment characteristics

Between 2007 and 2016, 37 patients with 43 lesions were treated with SBRT at our institute. Patient and tumor characteristics are summarised in Table 1. Seventeen lesions were identified as intrahepatic (IHCC) and 26 as extra-hepatic CCC (EHCC). Twenty six patients had a primarily inoperable disease, three patients were treated for positive margins and eight patients were treated for locoregional relapse after resection. Four patients had distant metastases, prior to SBRT, which were treated with chemotherapy (*n* = 3) or Radiofrequency Ablation (RFA, *n* = 1). These patients were treated either as oligometastatic or for oligoprogression. Twenty one patients (57%) had biliary stents prior to treatment. The median bilirubin concentration before treatment was 0.8 mg/dl (range: 0.2-21). Twenty patients had FDG-PET/CT prior to treatment as part of the initial staging or treatment planning. The median tumor diameter was 4.9 cm (range 2-18) with a median PTV volume of 124 cm^3^ (range 9-1356) and a median liver volume of 1576cm^3^ (range 843-3355).

**Table 1:** Patient and treatment characteristics

Parameter	Nr
A. Patient and tumor characteristics	
Age (years) Median (range)	67 (36-87)
Gender	
Male	24 (56%)
Female	19 (44%)
Tumor location ^a^	
IHCC	17 (40%)
EHCC	26 (60%)
Treatment	
Primary inoperable	26 (70%)
Recurrent	8 (22%)
Positive margins	3 (08%)
Prior therapies	
Resection	12 (32%)
Chemotherapy	6 (16%)
Gemcitabine/Oxaliplatin	1
Gemcitabine	1
Capecitabin	1
5-FU	1
Therapies after SBRT	
Chemotherapy	13 (35%)
TACE	1 (03%)
CA 19-9 (U/ml) Median (IQR)	149 (20-499)
Bilirubin (mg/dl) Median (range)	0.8 (0.5-21)
GTV Diameter (cm)^a^ Median (IQR)	4.9 (3.4-8.2)
PTV Volume (cm^3^)^a^ Median (IQR)	124 (60-329)
B. Treatment characteristics	
Prescribed dose (Gy)^a^	
Median (IQR)	45 (38-48)
Maximum point dose (Gy)^a^	
Median (IQR)	51 (44-58)
Mean PTV dose (Gy)^a^	
Median (IQR)	47 (42-51)
EQD2_10_ (Gy)^a^	
Median (IQR)	56 (47-61)
EQD2_10_ Maximum point dose (Gy)	
Median (IQR)	65 (59-85)
EQD2_10_ Mean dose (Gy)	
Median (IQR)	63 (53-82)

*IHCC* Intrahepatic cholangiocarcinoma, *EHCC* Extrahepatic cholangiocarcinoma, *5-FU* 5-Fluoruracil, *IQR* interquartile range, *EQD2* equieffective doses in 2 Gy (α/β = 10)

^a^per lesion

Median prescription dose was 45 (38-48) Gy corresponding to a median EQD2_10_ of 56 (range 25-61) Gy using a median dose per fraction of 4 Gy (range 4-12.5 Gy) in 3 to 12 fractions. Sixteen patients were treated with simultaneous integrated protection (SIP). Treatment characteristics are summarised in Table 1.

### Toxicity

Overall treatment was well tolerated. Three patients (9%) developed a Grade III bleeding. The first patient developed a gastric bleeding 4.3 months after SBRT and was treated with argon plasma coagulation. The dose maximum (D_max_) at the stomach was 45.6 Gy in 12 fractions, the Dose at 0.5cm^3^ (D_0.5_) was 43.7 Gy and at 5 cm^3^ (D_5_) was 40 Gy. The second patient was diagnosed with massive progression with ascites and peritoneal dissemination 3.7 months after SBRT and developed gastrointestinal bleeding. The patient refused esophagogastroduodenoscopy und thus the exact location of the bleeding could not be identified. The patient was under oral coagulation due to a pulmonary embolism. This patient died shortly after refusing any medical intervention. The maximum point dose D_max_ was 39.4 Gy in 12 fractions, the D_0.5cm_^3^ was 25,3 Gy and at 5cm^3^ D_5cc_ was 21Gy. The dose at the stomach was moderate so that one could hypothesize that the bleeding was caused due to portal hypertension because of ascites due to massive progression. The last patient developed a duodenal ulceration with bleeding after frequent biliary stenting due to tumor progression 12.6 months after SBRT. D_max_ at the duodenum was 25 Gy in 10 fractions. Institutional dose constraints for stomach and duodenum are shown in Table 2 including conversion to EQD2_3,_ using an α/β = 3 to account for late reacting normal tissue. None of the delivered doses to the specific OARs exceeded the institutional dose constraints. Seven patients developed a cholangitis, one due to a local progression which required stenting and all other patients because of a stent dysfunction which resolved after stent replacement.

**Table 2:** Correlation of toxicity with the maximum point dose delivered at the OAR and the institutional constraints

Toxicity	Nr of Fractions	OAR	Dmax (Gy) delivered	EQD2_3_ (Gy) delivered	Dmax constraint (Gy) for the OAR	EQD2_3_ (Gy) constraint
Gastric bleeding	12	stomach	45.6	62	47.4	65.8
GI bleeding	12	stomach	39.4	49.5	47.4	65.8
Duodenal ulceration	10	duodenum	25	27.5	44.3	65.8

*OAR* organ at risk, *EQD2*_*3*_ equieffective doses in 2 Gy (α/β = 3), *GI* gastro-intestinal, *Dmax* maximum point dose

### Local control and patterns of failure

**Fig. 1:**
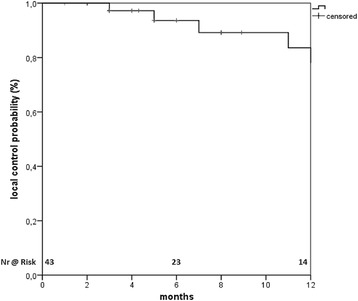
Local control from the start of radiotherapy

**Fig. 2:**
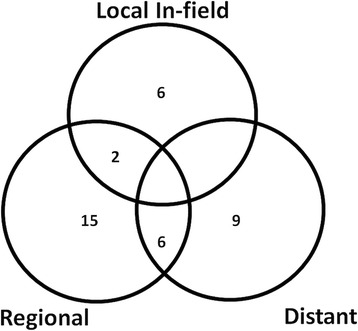
Venn diagram showing the patterns of relapse

The median follow up for patients alive was 24 months. The local control (LC) at 1 year was 78% and 58% at 2 years (Fig. 1). Median progression free survival (PFS) was 9 months (95% CI 2.8-15.1) from SBRT start, with a PFS at 1 and 2 years of 47% and 19%, respectively. Eight lesions progressed in field. Twenty one patients progressed in the liver but out of field (three of them with synchronous local lymph nodes) and 15 progressed distantly (Fig. 2). None of the regional progressions were marginal recurrences. Sites of distant progression were lung (*n* = 2), spleen (*n* = 1), peritoneal dissemination (*n* = 9), bones (*n* = 3). Thirteen patients were treated with chemotherapy after SBRT and one was treated with TACE. None of the variables tested (Table 3) correlated with local control, neither biological equivalent dose (BED) more than 100 Gy or 80.5 Gy as proposed by Tao et al. [12] or above the median at the encompassing isodose, at the maximum point dose or at the mean PTV.

**Table 3:** Univariate analysis for local control and overall survival

	Local control		Overall survival	
Variable	HR (95% CI)	*p*	HR (95% CI)	*p*
Location§	0.554 (0.111-2.772)	0.472	0.847 (0.4364-1.970)	0.700
GTV diameter (cm)	0.809 (0.613-1.068)	0.135	1.005 (0.900-1.122)	0.933
CA 19-9 (U/ml)	1.000 (1.000-1.001)	0.506	1.000 (1.000-1.000)	0.113
Bilirubin (mg/dl)	1.003 (0.838-1.201)	0.972	0.970 (0.861-1.093)	0.618
PTV Volume (cm^3^)	0.997 (0.992-1.001)	0.163	1.000 (0.999-1.002)	0.892
Prescribed dose (Gy)	0.749 (0.461-1.217)	0.243	0.994 (0.938-1.052)	0.823
Maximum dose (Gy)	0.997 (0.907-1.094)	0.940	0.983 (0.939-1.029)	0.455
Mean dose (Gy)	1.060 (0.952-1.180)	0.286	0.991 (0.937-1.048)	0.750
EQD2_10_ prescribed (Gy)	1.007 (0.938-1.081)	0.844	0.984 (0.944-1.025)	0.440
EQD2_10_ Maximum (Gy)	0.975 (0.940-1.012)	0.184	0.996 (0.977-1.017)	0.723
EQD2_10_ Mean (Gy)	0.971 (0.933-1.012)	0.148	0.993 (0.973-1.013)	0.474

### Survival outcomes

**Fig. 3:**
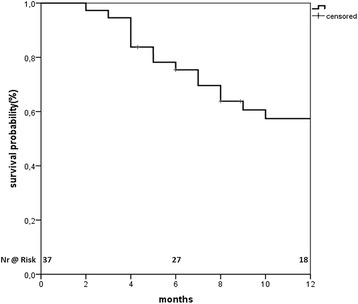
Overall survival from the start of radiotherapy

The median overall survival (OS) was 14 (95% CI: 7.8-20.2) months from start of SBRT and 22 (95% CI: 17.5-26.5) months from diagnosis. The OS from SBRT at 1 and 2 years was 57% and 25% (Fig. 3), respectively.

Eighteen patients died due to tumor progression, seven of unknown causes and two due to other causes. None of the patients died from therapy related cholangiosepsis. One developed a liver abscess, which was not associated with SBRT as it developed more than 3.5 cm from the edge of the PTV, after a biliary stent replacement 20 months after SBRT and died due to a bacterial peritonitis. Patients with prior metastases had a worse survival (14 vs 4 months, *p* = 0.2) but not statistically significant. Furthermore neither pre-treatment chemotherapy, nor pre-treatment CA 19-9 or bilirubin concentrations correlated with the OS on univariate and multivariate analysis.

## Discussion

Our institution has previously reported outcomes on the role of SBRT [22, 23]. In this study, we present the results of SBRT in both IHCCC and EHCCC in one of the largest series reported for CCC and especially for EHCC (Table 4).

**Table 4:** Review of literature on SBRT

Authors	Study	Localization	Nr. of Lesions	Nr. of Fractions	Total Dose (Gy)	LC @ 1 year	Median OS (months)	Late Toxicity
Tse [34]	P	IHCC EHCC	10 0	6	28-48	65%	15	1 biliary obstruction 1 bowel obstruction
Goodman [29]	P	IHCC EHCC	5 0	1	18-30	77%	28.6	None
Polistina [28]	R	IHCC EHCC	0 10	3	30^a^	80%^b^	35.5	1 ulceration 2 stenosis
Ibarra [35]	R	IHCC EHCC	11 0	3	22-50	55.5%	11	3 Grad 3
Barney [36]	R	IHCC EHCC	6 4	3-5	45-60	100%	15.5	1 Grade 3 biliary stenosis, 1 Grade 5 liver failure
Momm [22]	R	IHCC EHCC	0 13	10-12	32-56	78%	33.5	1 Grade 3 5 cholangitis
Weiner [37]	P	IHCC EHCC	12 0	5	40-55	91%§	13.2	1 hepatic failure§ 1 biliary stricture
Kopek [27]	R	IHCC EHCC	26 1	3	45	85%	10.6	6 ulcerations 3 stenosis
Mahadevan [30]	R	IHCC EHCC	31 11	3-5	24-45	88%	17	4 Grade 3 (ulceration, cholangitis, abscess)
Sandler [26]	R	IHCC EHCC	6 25	5	40	78%	15.7	5 Grade ≥ 3
Jung [25]	R	IHCC EHCC	33 25±	1-5	15-60	85%	10	6 Grade 3 (ulceration, cholangitis, stenosis, perforation)
Current	R	IHCC EHCC	17 26	3-12	21-66	78%	14	3 Grade ≥ 3

*R* retrospective, *P* prospective, *IHCCC* intrahepatic cholangiocarcinoma, *EHCCC* extrahepatic cholangiocarcinoma

^a^concurrent Gemcitabine

^b^local response ratio

± 5 patients treated with conventional fractionation with a stereotactic boost

§In this study SBRT was performed also in patients with hepatocellular carcinoma. LC and toxicities are reported for the whole group of patients including hepatocellular and cholangiocarcinoma

In the definitive and palliative setting, concurrent chemoradiation leads to a median OS of 2.2- 27 months and 3 y-years survival rates ranging from 6 to 73 months [24]. Local recurrence is the primary site of progression and dose escalation seems to be promising in terms of LC and OS. In a retrospective series using different fractionation regimes, Tao et al. could show that a BED greater than 80.5 Gy correlated with prolonged OS and LC (*p* = 0.017 and *p* = 0.04 respectively).These results, could not be reproduced in another retrospective study from Jung et al. [25] who treated patients using SBRT in 1-5 fraction. In his study a BED higher than 86 Gy did not correlate with better LC or survival (*p* = 0.4 and *p* = 0.1 respectively) which is in concordance with our findings. Furthermore, Jung et al. did not find any differences between patients treated for IHCCC (*n* = 33) vs EHCCC (*n* = 25) (*p* = 0.54) but they reported in 10% of the patients grade ≥ 3 complications such as duodenal and gastric ulceration and perforation as well as cholangitis and bile duct stenosis. Sandler et al. [26] reported 16% grade ≥ 3 toxicities in a retrospective analysis with 31 patients (IHCCC = 6, EHCCC = 25) treated with SBRT. Similar toxicities were also reported in a study of Kopek et al. [27] who treated 26 patients with Klatskin tumors. In this study, six patients developed a duodenal ulceration and 3 a duodenal stenosis (two of whom were among those with severe ulcers). They reported that the mean dose to 1 cm^3^ of duodenum (D 1 cm^3^) was significantly higher for patients developing grade ≥ 2 ulceration or stenosis at 37.4 Gy (83% of prescription dose) versus 25.3 Gy (*p* = 0.03) which corresponds to an EQD2_3_ of 115 Gy and 57.8 Gy, respectively. Kopek and co-workers suggested a V_21Gy_ ≤ 1 cm^3^ as a dose constraint for the duodenum in 3 fractions which corresponds to an EQD2_3_ of 42 Gy.

The increased toxicity in these studies is probably due to the large proportion of EHCCC included, because of the vicinity of these tumors with the duodenum. The above mentioned studies, together with the current one, are the largest reported series for EHCC (Table 4). In our series we had less late toxicities (3 cases of gastrointestinal bleeding), two of which could be explained due to other causes, such portal hypertension due to massive tumour progression and ascites and in the second case due to the manipulation after frequent biliary stenting. In both cases the institutional dose constraints were respected with a maximal EQD2_3_ point dose significantly less than 65.8 Gy. We could show a favourable toxicity profile probably due to a moderate fractionation and the use of simultaneous integrated protection (SIP). This concept is being further evaluated in a prospective trial.

To date, the role of SBRT is not clearly established, but there is emerging evidence that SBRT could lead to an improved OS, LC and symptom control. Preliminary results on SBRT [22, 28, 29] have reported promising median OS rates ranging from 28.6–35.5 moths. These results could not be confirmed in subsequent analyses which included a higher number of patients [25, 26, 30]. Although all of these studies including the current one have several limitations such as the retrospective design and small sample size median OS rates of 10–17 months and LC rates at 1 year of 55–100% seem highly promising in patients with inoperable or recurrent cholangiocarcinoma. In patients who receive chemotherapy receipt of radiotherapy was associated with improved survival [31]. SBRT can be well integrated to systemic chemotherapy with minimal interruption delivering an effective local treatment. Furthermore, radiotherapy does not show relevant impairment of the quality of life [32, 33] with the only observed deficits being temporary worsening of appetite and fatigue. Patients with advanced CCC and higher bilirubin concentrations are not candidates for chemotherapy and the only alternative is best supportive care. In our study those patients had a median OS of 12 months, higher than the rest, probably also due to patient selection bias, yet with a certain profit from SBRT in comparison to best supportive care. Stereotactic body radiation therapy is well tolerated and warrants further evaluation.

## Conclusion

Patients with cholangiocarcinoma, who are not candidates for surgical resection, have a dismal prognosis, and may benefit from locally ablative techniques such as SBRT. SBRT is a local treatment option with an acceptable toxicity profile, allowing its integration into multimodal treatment concepts. Prospective trials to validate these findings are underway.

## Abbreviations

CBCT: Cone beam computed tomography
CCC: Cholangiocarcinoma
CT: Computed tomography
CTCAE: Common terminology criteria for adverse events
dCC: Distal Cholangiocarcinoma
DEB-TACE: Drug-eluting bead trans-arterial chemoembolization
EHCC: Extrahepatic cholangiocarcinoma
EQD2: Equivalent dose in 2 Gy fractions
ERCP: Endoscopic retrograde cholangiopancreatography
FDG PET/CT: Fluorodeoxyglucose positron emission tomography
GTV: Gross tumor volume
HAI: Hepatic arterial infusion
ICRU: International commission on radiation units and measurements
IHCC: Intrahepatic cholangiocarcinoma
IMRT: Intensity modulated radiotherapy
ITV: Internal target volume
LC: Local control
MRCP: Magnetic resonance cholangiopancreatography
MRI: Magnetic resonance imaging
NCCN: National comprehensive cancer network
NCI: National Cancer Institute
OAR: Organs at risk
OS: Overall survival
pCC: Hilar/Perihilar cholangiocarcinoma
PDT: Photodynamic therapy
PTV: Planning target volume
RECIST: Response evaluation criteria in solid tumors
RFA: Radiofrequency ablation
SBRT: Stereotactic body radiotherapy
SIP: Simultaneous integrated protection
TACE: Trans-arterial chemoembolization
TARE: Transarterial radioembolization
